# Annual Recurrence of the Critically Endangered Bowmouth Guitarfish (*Rhina ancylostomus*) in Djibouti Waters

**DOI:** 10.3390/biology12101302

**Published:** 2023-10-02

**Authors:** Ginevra Boldrocchi, David Robinson, Simone Caprodossi, Emilio Mancuso, Moussa Omar, Jennifer V. Schmidt

**Affiliations:** 1 Department of Human Sciences, Innovation and Territory, University of Insubria, Via Valleggio 11, 22100 Como, Italy; 2 Sundive Research, Byron Shire, NSW 2481, Australia; david@sundive.com.au; 3 Marine Megafauna Foundation, West Palm Beach, FL 33411, USA; scaprodossi@hotmail.com; 4 Verdeacqua—Istituto per gli Studi sul Mare, Via Mac Mahon 33, 20155 Milan, Italy; emilio.mancuso@verdeacqua.org; 5 Centre d’Etude et de Recherche de Djibouti, Route de L’aéroport, Djibouti P.O. Box B.P.486, Djibouti; assoum_omar42@yahoo.fr; 6 The Shark Research Institute, Princeton, NJ 08540, USA; whalesharkjen@gmail.com

**Keywords:** *Rhina ancylostomus*, Gulf of Aden, shark, ray, wedgefish, guitarfish, Indian Ocean, rhino rays

## Abstract

**Simple Summary:**

One of the least understood and most threatened groups of elasmobranchs is the Rhinopristiformes, the guitarfishes, wedgefishes, and sawfishes. Numbering more than 60 species, this order includes a high percentage of Critically Endangered and Endangered species, as designated by the International Union for the Conservation of Nature. There is a dearth of data on these species due to sightings being infrequent and unpredictable. Globally, Rhinopristiformes are both directly targeted for their meat and large fins and are caught as bycatch in their near-shore habitats. We report here a consistent and predictable long-term presence of the Critically Endangered bowmouth guitarfish (*Rhina ancylostomus*) in the Gulf of Tadjoura, Djibouti. This species has been reliably encountered during diving and whale shark research activities (November–February) over seven seasons, indicating long-term site fidelity of *R. ancylostomus* within the area. Currently receiving minimal legal protection and facing unknown fishing pressures and ecological risks, these animals and their habitat should be prioritized for research and conservation.

**Abstract:**

The bowmouth guitarfish (*Rhina ancylostomus*) is among the most endangered of marine vertebrates, and evidence of severe declines and localized extinctions has been reported. Yet its life history and ecology suffer from a lack of scientific attention due to the scarcity and unpredictable movements of the species. By collecting opportunistic records from 2015 to 2023 during diving activities, this study describes for the first time the occurrence of a predictable aggregation of *R. ancylostomus* in the Gulf of Tadjoura (Djibouti). These data provide a key record of this species in the area, whose presence is strongly associated with sandy seabeds and corals at approximately 35 m depth. Based on the opportunistic sightings of *R. ancylostomus* during diving activity, Ras Eiro and Ras Korali appear to be the currently known core habitats for this species and may serve as breeding or feeding grounds. Overall, our data show that the Gulf of Tadjoura is a globally important conservation hotspot, and therefore its protection should be prioritized.

## 1. Introduction

The bowmouth guitarfish (*Rhina ancylostomus*) is a shark-like ray belonging to the order Rhinopristiformes. It occurs in the shallow coastal waters of the Indo-Pacific. It is listed as Critically Endangered on the IUCN Red List of Threatened Species, having undergone declines of > 80% across its range in the last 45 years [1,2]. 

*Rhina ancylostomus* and wedgefishes in general are heavily utilized for their meat, which is a source of protein for many coastal communities in tropical countries [2,3]. Wedgefishes are also traded for their large fins, considered the best quality for human consumption, placing them among the most highly valued in the international fin trade [3,4]. The fins of *R*. *ancylostomus* can fetch up to USD 964/kg on the international market. In addition, recent findings show that *R*. *ancylostomus* are harvested for their thorns, which are believed to carry protective powers in Thailand [5]. Wedgefishes, together with sawfishes and guitarfishes, have been identified as being among the most endangered marine vertebrates, and evidence of severe declines and localized extinctions has been reported [3,6].

Its high economic and food value represents a key extinction risk factor for *R*. *ancylostomus*, yet the life history and ecology of this species suffer from a lack of scientific knowledge [3]. *Rhina ancylostomus* is known to inhabit small bathymetries, from inshore to depths of at least 70 m, and it is mainly associated with soft-bottom habitats [1]. Soft-bottom habitats in shallow waters are generally more exposed to fishing activities than rocky or reef habitats, where the risk of gear loss or damage constrains fishing. This further contributes to *R*. *ancylostomus* habitat-related extinction risk factors [3]. Effective conservation biology depends on understanding the occurrence and distribution of threatened species to design appropriate conservation measures. In the case of *R*. *ancylostomus*, a detailed description of its presence, distribution, and habitat use at local and global levels is lacking. As a matter of fact, at present, the basic information on wedgefishes in general comes mainly from fisheries catch [7,8] or from unpredictable and sporadic encounters limited to single individuals [9]. 

Within the Gulf of Aden, the Gulf of Tadjoura (Djibouti) represents a hotspot of marine biodiversity due to the confluence of warm waters from the Red Sea and cooler water from the Somali and Arabian areas, which creates a productive marine ecosystem [10]. Indeed, several endangered and elusive species have already been reported in these waters: the whale shark, *Rhincodon typus* [11,12]; the scalloped hammerhead shark, *Sphyrna lewini*; the Indo-Pacific leopard shark, *Stegostoma tigrinum* [13]; the killer whale, *Orcinus orca* (unpublished records); and the Indian Ocean humpback dolphin, *Sousa plumbea* [14]. The Gulf of Tadjoura also hosts several threatened marine turtle species, including the green, *Chelonia mydas*, hawksbill, *Eretmochelys imbricata*, leatherback, *Dermochelys coriacea*, and loggerhead, *Caretta caretta*, turtles [10,15]. 

*Rhina ancylostomus* has been formally reported in the Gulf of Aden [2], but the occurrence of this species in the Gulf of Tadjoura has not been investigated. This study provides evidence for the first predictable aggregation site for the species, sourced from key initial records on the occurrence of *R*. *ancylostomus* in the Gulf of Tadjoura. This information begins the process of identifying core habitat areas, with the hope of stimulating future research on where to base species management plans.

## 2. Materials and Methods

**Figure 1:**
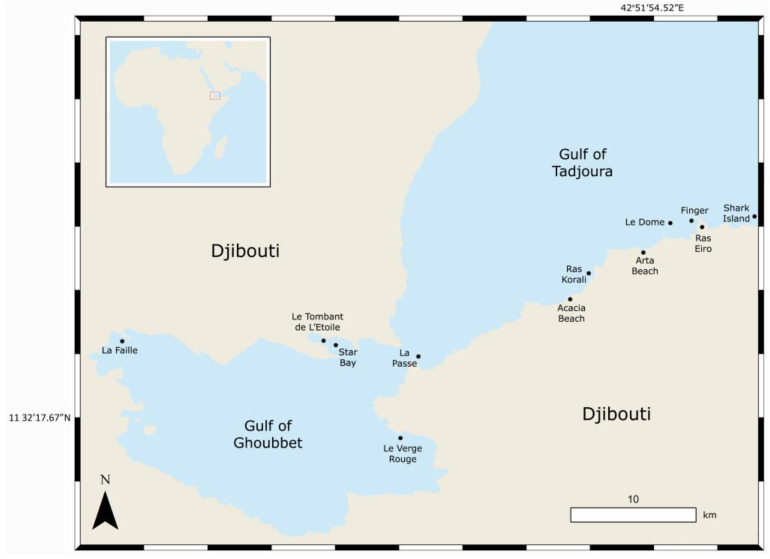
Map showing the surveyed dive sites within the Gulf of Tadjoura and Gulf of Ghoubbet.

This study was conducted in the coastal waters of the Gulf of Tadjoura ( 11°42′13″ N, 43°03′30″ E) and of Ghoubbet (11°31′55″ N, 42°36′16″ E), located in Djibouti, at the southern entrance to the Red Sea (Figure 1). Fieldwork was conducted from 2015 to 2023 as a side project during the whale shark aggregation season, between November and February on board a research vessel. Data were opportunistically collected during 7 sampling seasons, excluding 2020–2021 when no fieldwork was carried out due to the COVID-19 pandemic. Each cruise started and ended in Djibouti city and lasted one week, maintaining the same route and schedule each week. From Djibouti port, the boat moved to Ras Eiro (11°35′53″ N, 42°50′57″ E), where two diving sites were visited each week: *The Finger* and *The Dome* (Figure 1). The ship next visited Ras Korali (11°34′06″ N, 42°46′34″ E), where information was collected on another dive site, *Ras Korali*. The ship visited the dive site *La Passe* at the mouth of the Gulf of Ghoubbet and then entered the Ghoubbet and anchored at Star Bay (11°33′45″ N, 42°38′47″ E), where another dive site was sampled: *Le Tombant de L’Etoile* (Figure 1). From this bay, the vessel visited *La Faille* (11°35′09″ N, 42°32′00″ E), and then *La Vierge Rouge*, before returning via Star Bay, Ras Korali, and Ras Eiro. Prior to arrival at Djibouti city, the boat stopped at Shark Island (11°35′29″ N, 42°53′37″ E), where the last dive took place (Figure 1). Each diving site was visited in the morning, at around 7 am, before whale shark monitoring sessions, once per week, except for the dive site *Ras Korali*, which was monitored twice per week. Each dive lasted approximately 50 min.

For each encounter of *R*. *ancylostomus*, the following information was collected: date, location, time, and depth. Video and/or photo evidence was collected to ensure the correct species recognition, unless the sighting was by a researcher or trained dive guide. 

## 3. Results and Discussion

A total of 23 sightings of *R*. *ancylostomus* have been opportunistically recorded in seven sampling seasons (November–February). Apart from one snorkeling encounter near the Djibouti port, all records were collected during diving activities (Table 1). Of the 21 records that provided a precise dive location, 52.4% were recorded at *The Dome* (Ras Eiro) from 30 to 40 m depth, 42.9% at *Ras Korali*, at approximately 35 m, and 4.8% at *Shark Island* (Table 1) at 20 m depth. Additionally, the Critically Endangered Halavi guitarfish, *Glaucostegus halavi*, was recorded on a single dive at *Ras Korali* in 2016.

**Figure 2:**
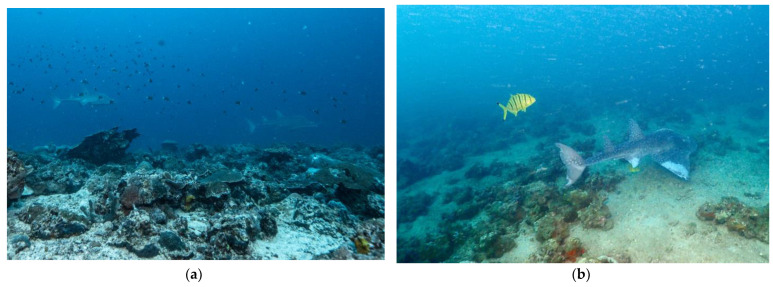
Individuals of *Rhina ancylostomus* encountered in Djibouti: (**a**) *R. ancylostomus* sighted in Ras Eiro (*The Dome*) in January 2017; (**b**) *R. ancylostomus* sighted at *Ras Korali* in December 2018.

The high percentage of sightings registered at *The Dome* and *Ras Korali* suggests that the area surrounding these dive sites can be considered an important habitat for this species in Djibouti and may also be for *Glaucostegus halavi*. Habitat preference of the shark-like batoids reported up to now includes soft-bottom habitats at < 50 m depth in warm temperate to tropical coastal waters [3]. Consistently, in the Gulf of Tadjoura, *R*. *ancylostomus* is strongly associated with sandy seabeds and corals at approximately 35 m depth, as at *The Dome* and *Ras Korali* (Figure 2). The dive sites where this species was not found (e.g., *La Passe* and *La Faille*) are either affected by strong currents or characterized by rocky habitats. 

**Figure 3:**
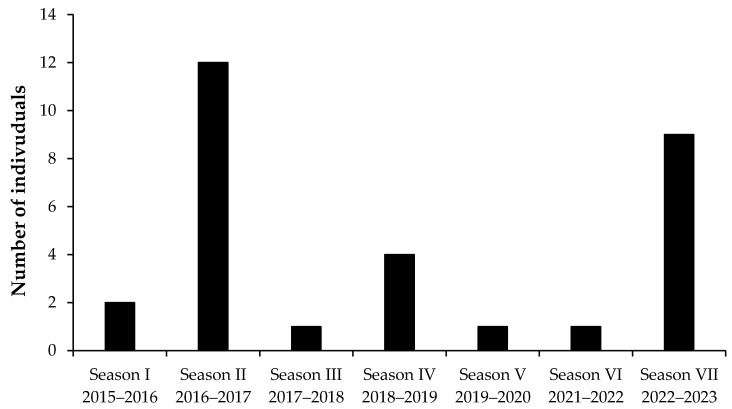
Temporal trend in seasonal opportunistic sightings of *Rhina ancylostomus* carried out in the Gulf of Tadjoura in 2015–2023.

Temporal analyses of the opportunistic sightings of *R*. *ancylostomus* in the Gulf of Tadjoura revealed a stable presence in the area (Figure 3). Although only three diving boats operate in the Gulf, *R*. *ancylostomus* have been regularly sighted during each diving season (November–February). During the 2019–2020 season, only one animal was sighted. In November 2019, Djibouti was affected by a severe flood, with almost 300 mm of rain recorded in Djibouti city, more than three times the annual average. The flood brought soil erosion and sewer discharge into the Gulf of Tadjoura coastal waters, which probably affected the *R*. *ancylostomus* presence and/or our capabilities to detect animals during dives. 

Despite the high-value meat and fins of *R*. *ancylostomus* [3,4], local fish market surveys during the study period did not document the presence of this species. Djibouti has few large-scale commercial fishing operations, with most activities confined to artisanal fishing. Typical catch profiles are composed mainly of Carangidae (21%), *Lethrinus* spp. (15%), Sphyraenidae (13%), *Scomberomorus* spp. (11%), *Lutjanus* spp. (10%), miscellaneous (10%), and *Thunnus* spp. (7%) [16,17]. With regards to condrichthyan species, *Carcharhinus amblyrhynchos*, *C*. *leucas*, *C*. *limbatus*, and *Sphyrna lewini* are the most commonly landed shark species [18]. No quantitative stock assessment or fishery indicators of status are currently available for *R*. *ancylostomus*, or for wedgefishes in general, in the Indian Ocean [19]. However, anecdotal evidence and historical catch data at various locations across its distribution range indicate localized population depletion [4]. For instance, in the Arabian Sea and adjacent waters, evident changes in the landings of shark-like batoids and the number of adult specimens have been reported during a monitoring program [4]. In India, the landings of *R*. *ancylostomus* declined by 86% from 2007 to 2020, despite an increase in fishing efforts [2,4,19] since *R*. *ancylostomus* are caught in trawl net and gillnet fisheries, which are common in India, and rarely in hook and line fisheries [19]. Reduced fishing pressure in Djibouti, primarily at the subsistence level using hook and line [20], might be connected to the stable and long-term presence of this species in the Gulf of Tadjoura.

Although the Rhinopristiformes are thought to aggregate in the shallow coastal waters of a few sites [21], this is the first time that a predictable aggregation of *R*. *ancylostomus* has been reported, highlighting the importance of the Gulf of Tadjoura for this species. Indeed, to the best of the authors’ knowledge, this is the first time that multiple individuals (up to seven; see Table 1) were recorded at the same location over multiple years. Indeed, few data have been published on this species, and these are mainly from fisheries catches. It is therefore quite challenging to compare Djibouti encounter rates with other locations. In lagoonal and inter-reef waters of the Great Barrier Reef Marine Park, 2471 baited remote underwater video stations were deployed between March 2000 and May 2010 [22]. In ten years of monitoring, the authors reported only nine encounters with *R*. *ancylostomus*, which is exceedingly low compared to those found opportunistically from limited diving in the Gulf of Tadjoura. *R*. *ancylostomus* is reported to have a maximum size of 270 cm total length, with males maturing at 150–175 cm, while females mature at ~180 cm total length [1]. The size of the observed individuals was approximately 200 cm or more, indicating adult specimens. This, coupled with the lack of neonate and/or juvenile sightings, suggests that the diving sites of Ras Korali and Ras Eiro may serve as breeding or feeding grounds for this species. Further studies should be carried out to broaden the monitoring areas and survey period. 

## 4. Conclusions

The protection of endangered species is an important component of regional and global conservation actions, which aim at maintaining or restoring remaining populations to improve their conservation status. Several elasmobranch species recorded in the Gulf of Tadjoura are threatened with extinction. This study provides evidence of the regular presence of the Critically Endangered bowmouth guitarfish in Djibouti waters, and therefore, comprehensive and coordinated actions are urgently needed for its protection. As shown in multiple fisheries monitoring programs in the Gulf of Aden and nearby waters, this species is facing a decline in numbers. While opportunistic surveys did not document the presence of this species at the local fish market, direct catches remain an ongoing threat to this population, together with bycatch, pollution, growing tourism, and plans for greatly increased shipping through the Gulf. Our findings, in addition to previous elasmobranch studies that have been carried out in the area, indicate that within the Gulf of Tadjoura there are critical areas that host multiple species of threatened elasmobranchs. As conservation biology is increasingly moving in the direction of preserving critical habitats rather than individual species, the Gulf of Tadjoura should be considered at both national and international levels for strong protection. In this regard, the designation of an ISRA—Important Shark and Ray Area—(https://sharkrayareas.org/) (accessed on 28 September 2023) would stimulate further monitoring programs and facilitate appropriate conservation actions, potentially resulting in Marine Protected Areas and other forms of effective conservation. 

## Figures and Tables

**Table 1:** Total records of *Rhina ancylostomus* in the Gulf of Tadjoura between November and February in 2015–2023.

Date	Dive Site	Depth (m)	Family	Species	Photo/ Video	*n* Individuals	Source
4 November 2015	*Ras Korali*	20–25	Rhynidae	*Rhina ancylostomus*	yes	1	Trained Diver
16 November 2015	*Ras Korali/The Dome*		Rhynidae	*Rhina ancylostomus*	yes	1	Trained Diver
14 November 2016	*Ras Korali*	20–25	Glaucostegidae	*Glaucostegus halavi*	yes	1	Guest
20–26 November 2016	*Ras Korali*	20–25	Rhynidae	*Rhina ancylostomus*	no	1	Researcher
7–14 January 2017	*Ras Korali*	20–25	Rhynidae	*Rhina ancylostomus*	yes	1	Guest
1 January 2017	*The Dome*	30–35	Rhynidae	*Rhina ancylostomus*	yes	1	Trained Diver
13 January 2017	*The Dome*	30–35	Rhynidae	*Rhina ancylostomus*	yes	7+	Researcher
14 January 2017	*Ras Korali*	20–25	Rhynidae	*Rhina ancylostomus*	no	1	Researcher
14 January 2017	*The Dome*	30–35	Rhynidae	*Rhina ancylostomus*	no	1	Researcher
20 November 2017	*Ras Korali*	20–25	Rhynidae	*Rhina ancylostomus*	yes	1	Trained Diver
28 November 2018	*The Dome*	30–35	Rhynidae	*Rhina ancylostomus*	no	1	Trained Diver
29 November 2018	*The Dome*	30–35	Rhynidae	*Rhina ancylostomus*	no	1	Trained Diver
7 December 2018	*Shark Island*	24	Rhynidae	*Rhina ancylostomus*	yes	1	Guest
December 2018	*Ras Korali*	20–25	Rhynidae	*Rhina ancylostomus*	yes	1	Trained Diver
26 December 2019	*Ras Korali*	20–25	Rhynidae	*Rhina ancylostomus*	yes	1	Trained Diver
28 December 2021	*The Dome*	30–35	Rhynidae	*Rhina ancylostomus*	yes	1	Guest
2 December 2022	*The Dome*	30–35	Rhynidae	*Rhina ancylostomus*	yes	1	Trained Diver
2 December 2022	*Ras Korali*	20–25	Rhynidae	*Rhina ancylostomus*	yes	2	Trained Diver
18 December 2022	*The Dome*	30–35	Rhynidae	*Rhina ancylostomus*	no	1	Trained Diver
End/December 2022	*The Dome*	30–35	Rhynidae	*Rhina ancylostomus*	yes	1	Trained Diver
End/December 2022	*The Dome*	30–35	Rhynidae	*Rhina ancylostomus*	no	1	Trained Diver

## Data Availability

Data are contained within the article.
